# Integrated Dental Practice Management in Romania: A Cross-Sectional Case–Control Study on the Perceived Impact of Managerial Training on Efficiency, Collaboration, and Care Quality Dental

**DOI:** 10.3390/healthcare13131631

**Published:** 2025-07-07

**Authors:** Georgiana Ioana Potra Cicalău, Liana Todor, Roxana Alexandra Cristea, Ramona Hodișan, Olivia Andreea Marcu, Ioan Andrei Țig, Lucia Georgeta Daina, Gabriela Ciavoi

**Affiliations:** 1 Department of Dental Medicine, Faculty of Medicine and Pharmacy, University of Oradea, 410068 Oradea, Romania; cicalau.georgiana@uoradea.ro (G.I.P.C.); liana.todor@uoradea.ro (L.T.); itig@uoradea.ro (I.A.Ț.); gciavoi@uoradea.ro (G.C.); 2 Department of Preclinical Disciplines, Faculty of Medicine and Pharmacy, University of Oradea, 410068 Oradea, Romania; omarcu@uoradea.ro; 3 Department of Psycho-Neurosciences and Recovery, Faculty of Medicine and Pharmacy, University of Oradea, 410081 Oradea, Romania; lgdaina@uoradea.ro

**Keywords:** dental practice management, leadership, dentist, manager, dental disciplines collaboration

## Abstract

**Background/Objectives:** The effective management of dental practices is increasingly recognized as a key factor in ensuring high-quality care, efficient operations, and interdisciplinary collaboration. While many dentists assume managerial responsibilities, formal training in healthcare or dental practice management may influence the quality of these practices. This study aims to evaluate the differences in organization, efficiency, and quality of care between dental clinics managed by dentists who have completed management training and those who have not. It also explores dentists’ knowledge and attitudes regarding dental practice management. **Methods:** A cross-sectional, observational, case–control study was conducted between 14 April and 14 May 2023, using an online questionnaire distributed to licensed dental practitioners in Romania. A total of 136 dentists participated, divided into a study group (*n* = 60), who had completed management courses and a control group (*n* = 76) who had not. Descriptive statistics and comparative analyses (*t*-tests, Chi-square tests) were performed using SPSS version 24, with significance set at *p* < 0.05. **Results:** Dentists with managerial training demonstrated greater implementation of strategic planning, financial performance monitoring, quality management, and use of digital tools. They also reported higher collaboration with interdisciplinary professionals—orthodontist 76.7% in the study group vs. 63.2% in the control group, medical assistant 78.3% in the study group vs. 47.4% in the control group, front desk 43.3% in the study group vs. 18.4% in the control group; better delegation of tasks—61.7% in the study group vs. 27.6% in the control group; and greater concern for team development—95% in the study group vs. 71% in the control group; and patient rights—81.7% in the study group vs. 75% in the control group. Significant differences (*p* < 0.05) were noted in management practices, opinions about the optimal manager for a dental practice, and the use of software tools. **Conclusions:** Managerial training equips dentists with critical skills for enhancing operational efficiency and care quality. Integrating management education into dental curricula and continuing professional development can substantially improve the sustainability and performance of dental practices.

## 1. Introduction

Over time, numerous authors have proposed various definitions of management. Hersey and Blanchard define management as working with individuals or groups to accomplish organizational objectives [1]. In contrast, Brech states that management involves assuming responsibility for decisions, planning, and regulating the activities of individuals working toward a shared goal, ensuring that outcomes are both efficient and economical [2]. Henri Fayol emphasized that management encompasses forecasting and planning, organizing, leading, coordinating, and controlling [3,4].

Management functions encompass the key activities managers use to achieve organizational goals by effectively leveraging human resources. According to Henri Fayol, these functions include planning, organizing, leading, coordinating, and controlling. Planning involves forecasting and preparing actions to ensure efficiency. Organizing defines the structure and roles necessary to meet objectives. Leading deals with staffing and performance management. Coordination focuses on motivating and communicating with employees to align efforts. Control monitors performance against standards to enable continuous improvement [3,4].

In the current environment, the roles of managers have expanded significantly, transcending traditional functions to encompass adaptation to rapid workplace changes, fostering collaboration, and integrating emerging technologies. According to a study published by Wang et al., managers must develop new competencies to meet digital challenges, such as adopting innovative technologies and managing teams in virtual settings [5]. Additionally, research highlights the importance of managerial support in promoting flexible work arrangements and work–life balance—factors that have become essential in the post-pandemic era. These developments underscore the necessity for managers to adopt an empathetic and adaptable leadership style that can accommodate the diverse needs of employees [6].

An article published by Bolino et al. (2024) [7] and Béland et al. (2022) [8] explores how the COVID-19 pandemic has reshaped managerial practices, emphasizing the increasing demand for flexibility and innovation in leadership approaches. These perspectives suggest that effective managers must not only act as strategic leaders but also serve as change facilitators and advocates for employee well-being [7,8]. Accordingly, the recent literature stresses that managerial success in the contemporary era hinges on the ability to navigate digital complexity, support teams through organizational transitions, and promote a culture of continuous learning and adaptability.

Organizational management involves planning, coordinating, and controlling resources to achieve strategic goals, and its effectiveness today depends on leaders’ ability to leverage new technologies, motivate employees, and foster a flexible, collaborative environment that adapts to rapid changes and supports continuous learning [7]. Managing a dental practice involves applying general management principles within the unique context of healthcare delivery combined with running a business. Effective management includes planning services, organizing the team and schedules, ensuring adequate material and financial resources, and monitoring both clinical and financial outcomes. Dentistry particularly emphasizes clear communication with patients, high-quality medical care, and adherence to health regulations. Often led by the dentist, management also requires handling human resources, marketing, supplier relationships, and adopting technological innovations. Today, the success of a dental practice depends not only on clinical skills but also on the ability to sustainably manage a patient-focused business [9].

The financial management of a dental practice plays a crucial role in maintaining economic stability and supporting sustainable growth. It involves the development of a detailed budget, monitoring cash flow, efficient management of receivables, and the implementation of rigorous internal controls to prevent fraud and accounting errors. A clear separation between personal and business finances is essential, as is the careful tracking of costs associated with each service offered, in order to evaluate their profitability. Implementing sound accounting practices supports informed decision-making regarding investments, pricing strategies, and fiscal planning, thereby ensuring the long-term success of the practice [10].

Over the past decade, extensive research globally has focused on improving team effectiveness in healthcare through various interventions such as principle-based and simulation training, communication tools, and organizational redesign, with strong evidence supporting their positive impact on non-technical skills and team functioning [11]. In Romania, however, oral health policies and dental care delivery have faced systemic challenges—including limited funding and constrained access—that have hindered progress despite a sufficient number of practitioners. While international studies have advanced the understanding of team-based interventions, there remains a critical need to explore how these insights translate within the Romanian context, particularly given the unique interplay of cost, quality, and access to care in shaping oral health outcomes [11,12].

The organization and operation of dental practices in Romania are governed by legal frameworks that ensure safe service delivery and adherence to professional standards. Key regulations include Law No. 95/2006 on health reform, which outlines the establishment and authorization of dental practices, alongside Ministry of Health rules on hygiene and sanitation. The Code of Ethics for Dentists, approved by the College of Dental Physicians, sets ethical and professional guidelines. Dentists must obtain a membership certificate from the College, confirming they meet training and competence requirements per Law No. 95/2006, and are required to participate in continuing education to maintain their authorization [7]. In Romania, dental practices may be organized under three distinct legal forms: Individual Medical Practice (Cabinet Medical Individual, CMI), Limited Liability Company (Societate cu Răspundere Limitată, SRL), and Independent Natural Person (Persoană Fizică Independentă, PFI). Each legal form has specific implications for the administrative, fiscal, and professional structure of the practice [13].

Dentists are required to complete continuing medical education (CME) through accredited courses recognized by the College of Dental Physicians to maintain their license and keep their skills up to date. Additionally, those contracting with the National Health Insurance House (CAS) must follow specific regulations regarding service provision, payment, and reporting, while ensuring their practice meets infrastructure and equipment standards to offer care to insured patients [10].

Dental medicine includes multiple specializations that work closely together to ensure comprehensive and high-quality treatment for patients. Specialties such as orthodontics, oral surgery, periodontology, and prosthodontics collaborate to address various aspects of oral health. Interdisciplinary collaboration among dentists and other professionals—such as dental technicians, radiologists, administrative staff, and cleaning personnel—is essential to the success of a dental clinic. The dental technician contributes by creating customized prosthetics, the radiologist provides essential diagnostic imaging, administrative staff manage appointments and patient records, and cleaning staff maintain hygienic conditions. Effective management is critical for coordinating these activities, ensuring smooth team interactions, and optimizing workflow [14].

Effective management of complex health conditions like diabetes mellitus and periodontal disease benefits from a multidisciplinary approach, involving collaboration among healthcare professionals such as dentists, diabetologists, and other specialists to create personalized treatment plans, while complementary therapies—including various phytotherapeutic agents—can support conventional treatments by reducing inflammation and promoting healing [15,16,17,18,19,20,21,22,23,24,25,26]. Integrating psychologists into dental practices exemplifies a valuable multidisciplinary approach, as they can support patient care by managing dental anxiety through communication strategies and relaxation techniques, while also helping dental staff cope with work-related stress, thereby enhancing overall patient experience and team well-being [27,28,29].

The literature consistently indicates that differences exist between dental practitioners who have completed management training and those who have not. Therefore, the hypothesis is that significant differences are present between these two groups.

Aim of the Study

The purpose of this study is to analyze the differences between dental practices led by dentists who have completed a course in healthcare management or dental office management and those led by dentists without such training, from the perspective of organization, operational efficiency, and quality of services provided.

## 2. Materials and Methods

### 2.1. Study Design and Population

In the present research, we conducted a cross-sectional, observational, case–control study. The study targeted dental practitioners in Romania and was carried out through a questionnaire developed using Google Forms. The questionnaire, specifically designed for our study, was distributed via online platforms. This research represents a pilot study. The primary aim was to explore initial trends and assess the feasibility of our methodology, including the use of an original questionnaire. Further validation and expanded analysis are planned in future, larger-scale studies.

The study was conducted during the period of 14 April 2023, to 14 May 2023, and was reviewed and approved by the Research Ethics Committee of the Faculty of Medicine and Pharmacy, University of Oradea, receiving favorable opinion No. CEFMF/3 dated 13 April 2023.

The questionnaire comprised 27 questions (Supplementary Materials). Dentists were invited to participate voluntarily in this study, with the completion of the questionnaire serving as informed consent and agreement to participate. No personal identifying information, such as names or email addresses, was collected, and all responses remained anonymous to encourage honesty and transparency.

Section one—Demographic Information (questions 1–6): The first section included six questions covering: age, gender, organizational form of dental practice activities, location of the dental office, time from graduating and the dental specialist’s field of expertise.

Section two—Management of the Dental Practice (questions 7–21): The second section comprised 21 questions focused on various aspects of dental practice management, aiming to assess organizational, financial, and operational practices within dental clinics.

We used closed, single-choice format questions, and only the question addressing respondents age was an open-ended one. The average time required for completion was about 3–5 min.

This study was addressed exclusively to dentists, and upon completion, a total of 136 questionnaires were collected. Respondents were divided into two groups: the first group consisted of dentists who had previously attended a course in Healthcare Management or Dental Practice Management (hereafter referred to as the study group), and the second group included dentists who had not participated in such courses (referred to as the control group). As a result, the study group comprised 60 dentists, while the control group included 76 dentists. The total sample size required was calculated using the following parameters: significance level 0.05, power of the test 0.75, and effect size 0.5. The minimum number of observations in each group was 57.

### 2.2. Inclusion and Exclusion Criteria in the Study

In forming the study groups, we took into account the characteristics of the respondents necessary for determining the sampling method. Eligibility criteria were thus established and divided into general inclusion criteria, inclusion criteria for the study group, and inclusion criteria for the control group, as follows:

- General inclusion criteria:
  ○ dentists who expressed their willingness to participate in the study;
  ○ licensed dental practitioners;
  ○ residents, specialists, or senior dentists;
  ○ dentists aged between 24 and 80 years;
  ○ dentists of all genders;
  ○ dentists practicing in both urban and rural settings.
- Inclusion criteria for the study group
  ○ dentists who have completed courses in Healthcare Management or Dental Practice Management.
- Inclusion criteria for the control group
  ○ dentists who have not completed courses in Healthcare Management or Dental Practice Management.

### 2.3. Statistical Analysis

The data collected through the administered questionnaires were organized into a database using Microsoft Excel. Statistical analysis was conducted using SPSS version 24 (Armonk, New York, NY, USA), a software specialized in statistical processing. Descriptive statistics for quantitative variables are presented as means, while qualitative variables are described using population percentages. Differences between the two groups of respondents were assessed using specific statistical tests: the independent samples *t*-test for quantitative variables and the Chi-square test for qualitative variables. A significance level of *p* < 0.05 was considered statistically significant. Where results did not meet this threshold, the exact *p*-value is reported.

### 2.4. Limitations of the Study

The present study has certain limitations due to the relatively small number of respondents, which can be attributed to the limited willingness of medical personnel to complete online questionnaires. The reduced sample size may introduce potential errors in statistical interpretation.

## 3. Results

### 3.1. Demographic Characteristics of the Respondents

The demographic data of the participants included in the clinical study comprised age, gender, and the geographical location of the dental practice. Respondents in the present study were divided into three age groups: Group I represented by respondents aged between 24 and 39 years, Group II represented by people aged between 40 and 55 years, and Group III with respondents aged between 56 and 68 years.

The distribution of respondents according to average age does not differ significantly from a statistical point of view (*p* = 0.419). Age group I was more prevalent in the control group compared to the study group, while age group II was more represented in the study group compared to the control group. The fewest respondents were in age group III (Table 1).

The distribution of dentists by gender does not show statistically significant differences (*p* = 0.738). In both groups, female respondents were predominant. There were no statistically significant differences regarding the location of the dental practice (*p* = 0.16) (Table 1).

### 3.2. Professional Characteristics of the Respondents

Table 2 shows the distribution of doctors according to professional characteristics. There are no statistically significant differences between the two groups regarding the organizational structure of the dental practice (*p* = 0.185), the professional experience of the respondents included in the study (*p* = 0.069), and in the distribution of respondents according to professional qualifications (*p* = 0.411).

At a significance level of 0.05, significant differences were observed between the two groups regarding their responses about who is the best manager of a dental practice. The majority of respondents in the control group believe that the dentist is the best manager of the dental practice, whereas in the study group, most respondents consider that a specialized manager is the most suitable to manage a dental practice (Table 3).

### 3.3. Managerial Characteristics of the Respondents

When comparing the two groups in terms of the categories of collaborators in the dental practice, we observe that more than 50% of those in the study group collaborate with a dentist, orthodontist, endodontist, maxillofacial surgeon, dento-alveolar surgeon, medical assistant, dental technician, accountant, and cleaning staff. Statistical differences were observed between the two groups in collaboration with a dentist (*p* = 0.014), with medical assistants (*p* < 0.01), radiology technicians (*p* = 0.042), front desk staff (*p* = 0.002) and with managers (*p* = 0.046). In contrast, in the control group, over 50% collaborate only with an orthodontist, dental technician, accountant, and cleaning staff (Table 4).

No major differences are observed between the two groups in terms of their distribution based on responses regarding the tasks of the medical assistant in the dental practice; in all situations *p* > 0.05. The responses are nearly unanimous regarding the medical assistant’s tasks of sterilizing instruments and preparing the office and necessary materials (Table 5).

Regarding the responses concerning the management of the dental practice, we observe that respondents in the study group show greater concern for management methods and programs implemented to streamline processes within the dental practice, compared to the control group. Statistically significant differences were observed in the existence of concerns regarding employee training, possession of a dental practice management program, the existence of a strategic plan at the level of the dental practice, possession of liability insurance for the dental practice and designation of a person responsible for supplying the dental practice with dental materials (*p* < 0.01). (Table 6).

When comparing the modern management tools used in dental practices between the two groups, we observed a greater openness towards the use of management software (*p* = 0.004) and collaboration with specialized marketing firms (*p* = 0.005) among the respondents in the study group compared to those in the control group. Regarding the use of the internet and environmental elements in the dental practice, differences were observed between the two groups (Table 7).

## 4. Discussion

Following the statistical processing of the demographic characteristics of the dentists included in our study, we identified that the majority of them fall into the age group I (24–40 years), with only five respondents being in age group III (56–68 years). These results can be explained by the higher accessibility of young dentists to continuing medical education (CME) courses related to dental practice management. Another explanation could be the lack of management courses in previous years, particularly those dedicated to dental practice management, due to the epidemiological situation generated by the SARS-CoV-2 virus infection [30]. On the other hand, participation in management courses by dentists was also reduced due to the financial crisis in our country, with research by Murariu et al. (2019) demonstrating that 60.7% of dentists gave up on specialization courses as a financial recovery measure [31].

The literature shows that the opinions of dentists regarding the content of a dental practice management course can be extremely valuable and must be considered when designing and implementing such a course for dental practices [32,33].

Our research shows that the majority of respondents are female (61.8%). In a similar study, more responses were also obtained from women (63.3%) [34]. The percentage difference between the two genders can be explained by the greater openness of females towards completing the questionnaire. Furthermore, statistics show that among dentists in Romania, females are the majority (66.5%), further supporting our results [35].

Regarding the location of the dental practice, we found that the majority are located in urban areas (80.9%). The deficit of dental practices in rural areas can be explained by the lack of motivation among dentists to establish practices in rural areas, as well as the low demand for dental services from residents of these areas [36]. Recent studies show that in rural areas of Romania, there is one dentist for every 4600 residents, while in urban areas, there is one dentist for every 631 residents [37,38].

In terms of the organization of dental practices, limited liability companies (LLCs) predominate in the study group, while individual medical practices (IMPs) are the majority in the control group. When choosing the appropriate business structure for a dental practice, it is important to understand the legal strategies associated with each organizational form [39]. According to the American Dental Association, there are several organizational forms available, each with certain implications, advantages, and disadvantages [40]. In Romania, the most common forms of dental practice are individual medical practices (IMPs) and limited liability companies (LLCs), as shown in our study.

At the beginning of their careers, many dentists choose to establish individual medical practices (IMPs) due to lower bureaucratic demands, minimal startup costs, and simpler financial management. IMPs are taxed on profit, whereas limited liability companies (LLCs) are taxed on revenue. In our study, most respondents opted for IMPs, likely for their financial flexibility. However, IMPs may restrict professional activities to clinical practice alone. In contrast, dentists who have completed management training often choose LLCs, allowing broader professional involvement such as employing staff, participating in conferences, or engaging in academic and promotional activities. Both organizational forms are eligible for contracts with the National Health Insurance House (CNAS) and can subcontract specialists [41].

According to the 2014 Manual of Dental Practice, there were 6603 private dental practices and 7000 dental practices under contract with CNAS in Romania. Public dental services at that time were provided by only 1200 dentists [42]. In countries like France, the number of dentists working in the public system is comparatively higher, with a total of 2828 dentists in 2012, and in Italy, 3157 dentists [43,44].

The exact number of dentists working solely in the public sector is not known, as many also operate in private practice. In recent decades, public schools have been the main providers of public dental care, but their numbers have steadily declined [42]. Currently, Romania faces a shortage of dental practices in pre-university and university institutions. For instance, Bucharest has 137 school dental offices and 13 university practices, while Constanța has only 12 facilities where students can access CNAS-reimbursed services [45,46]. To promote oral health and community well-being, there are plans to implement preventive programs and increase the number of school dental offices, in accordance with Order No. 438/4.629/2021 on medical care for students at all educational levels [47].

In our study, a higher percentage of dentists in the study group had contracts with the National Health Insurance House (CNAS), enabling the provision of primary dental care. As noted by Da Rocha Mendes et al. (2021), ongoing professional development and management training are essential for improving primary dental services and should inform health policy strategies [48]. Polverini (2012) also emphasized the importance of integrating leadership development into dental education to support the future of oral healthcare systems [49].

The majority of respondents in the study group have over 10 years of professional experience, which suggests that with the accumulation of professional experience, dentists are more interested in participating in management courses. Our study results are similar to those of Wang et al. (2020), who suggest that the accumulation of practical skills in a dental career, involving age, professional qualifications, and leadership abilities, helps promote entrepreneurial intent, whereas a diploma focused on academic activity alone does not [50]. On the other hand, regarding professional training, most respondents in the study group are general dentists (46.7%) and specialist dentists (26.7%), suggesting that specialization does not influence participation in management courses, according to our study results (*p* = 0.411).

This research identified statistically significant differences between the two groups regarding the question of who would be the best manager of a dental practice. Respondents who have attended management courses consider that a specialized manager is the most suitable to lead and manage the dental practice, while dentists who have not attended such courses believe that the dentist themselves would be the best manager of the dental practice. Nazir et al. (2018) [34] showed that dentists who participated in dental practice management courses are significantly more likely to agree on the need for such a course for a successful dental practice. The development and implementation of such a course should be based on feedback from dentists to ensure evidence-based approaches [34].

Respondents from the study group who have attended management courses exhibit a higher percentage of collaborators in the following categories: dentist, orthodontist, pediatric dentist, prosthodontist, endodontist, maxillofacial surgeon, dental surgeon, medical assistant, radiology technician, front-office receptionist, manager, and cleaning staff, compared to the control group. According to Koyamata et al. (2022), interdisciplinary dental–medical collaboration develops professionals in the field based on the knowledge and skills acquired from experienced doctors, resulting in specialized treatment teams [51].

In both groups, the dental technician and accountant are the main collaborators of the dental practice. Good-quality communication between the dentist and dental technician is considered fundamental, as it influences the potential for high-quality prosthetic work [52,53]. Proper collaboration between the dental practice and dental laboratory provides the advantages of well-designed prosthetic work, a satisfied clinician, and a comfortable professional relationship between the dentist and dental technician. Alshiddi et al. (2014) developed a web application for communication between dental practices and dental laboratories, the Web Content Management System, which offers several advantages for both members of the team [54]. Azzopardi et al. (2020) [55] found that good communication between the dental clinic and dental laboratory has both strengths and weaknesses in the eyes of dental technicians. There is room for improvement in communication between these two professions [55]. Updating communication between the dental practice and the dental laboratory will save time, effort, and improve the quality of the final product. Moving to a paperless dental practice is recommended and may become mandatory when establishing a dental clinic in some countries. Choosing the appropriate web content management system by understanding individual requirements and software design will significantly improve communication and ensure long-term relationships between dental practices and dental laboratories [54].

The role of the accountant is to keep pace with this ever-changing environment, ensuring that they present the technology and tools that help the dentist streamline practice management, contributing to time savings and identifying business improvement strategies. The role of accountants for dentists will undergo a massive change in the coming years. Practices that will thrive will be those that have efficient financial management and perspectives for managing and maximizing profitability [56].

Additionally, findings suggest that there are gaps in the relationship between dental staff and primary healthcare providers. Interprofessional collaboration between dental healthcare providers and non-dental primary healthcare providers, especially in rural and isolated communities, could be improved by organizing regular meetings between practitioners from all health disciplines, providing oral health education to primary care providers, establishing and maintaining effective communication and referral pathways, and exploring a larger role for teledentistry [57].

The ambition to provide quality care for patients is driven by high performance through patient-focused teams. However, there is a need to reduce the gap between traditional practices and the new attitudes required for an efficient team to achieve such high ambition [53]. Therefore, healthcare organizations should aim to provide exceptional patient care by adopting a culture of broad teams, where certain values and principles are shared and communicated transparently between team members, including patients, who should be placed at the center of care [52].

Regarding the medical assistant’s tasks in a dental practice, respondents from both groups have similar opinions. They consider the following as the medical assistant’s tasks: scheduling patients, sterilizing instruments, preparing the necessary medical–legal documents for the therapeutic act, preparing the patient’s treatment record, preparing the office and required materials, and recording treatments in the consultation register and treatment sheet.

In dental practices, the medical assistant is often assigned multiple tasks that exceed the responsibilities outlined in their job description. Often, they take on the tasks of a receptionist or front-office staff in ultra-modern dental clinics. Kottek et al. (2021) [58] successfully implemented a care coordination program in a large dental organization in the United States by enhancing the competencies of front-office staff. This program involved a strong organizational commitment based on teams and positive feedback on care coordination between staff and managers. Improving the competencies of existing administrative staff with the necessary training to manage the needs of high-risk patients is a method that can be used to implement care coordination efforts in dentistry [58].

In the case of dentists who have not attended management courses, there is less concern for staff training and they lack management programs in a higher number compared to those in the study group (*p* < 0.01). Additionally, the existence of a strategic plan, a quality management plan, a risk management plan, or periodic financial performance analysis is rarer among dentists who have not taken management courses. Behavioral economics combines research from the fields of psychology, neurology, and economics to help staff understand how individuals make decisions in complex social and economic environments. The principles of behavioral economics are increasingly applied in healthcare. Scarbecz (2012) described how members of the dental team can use behavioral economics principles to improve patients’ oral health [59]. Ungureanu et al. (2014) stated that to achieve a higher degree of implementation, quality management activities should be better regulated, training should be offered by a professional associations of dentists, and dentists should receive support in selecting quality management tools that are suitable for their dental practice [60].

The growing demand for quality, patient-centered care requires healthcare professionals to adopt a collaborative, team-based approach. Babiker et al. (2014) emphasize that this is achieved by placing the patient at the center and fostering shared values within the team [53]. Motivating team members through practical strategies is essential to reaching goals and overcoming challenges. In dental practices, the benefits of management training are supported by transformational leadership theory, which shows how trained dentists can inspire teams and improve efficiency through vision and motivation, and by Kolb’s experiential learning theory, which explains how management skills are applied through reflection and real-world adaptation [61,62]. Consequently, proper communication with collaborators and patients is necessary for making decisions that guide us toward success. Problem-solving and positive communication contribute to increasing staff trust, improving relationships, maximizing cooperation, minimizing misunderstandings, and significantly reducing existing stress [63].

Sustainability in dentistry is increasingly important, requiring environmentally responsible practices and education to support global sustainability goals [64,65]. The modernization of dental practices, especially the use of disposables, calls for a re-evaluation of sustainability concepts [66]. Understanding dentists’ attitudes can help shape educational programs for current and future practitioners. Țâncu et al. (2023) reported moderate awareness of sustainability among dentists, underscoring the need for targeted training [67]. In the control group, only 31.6% of dentists had liability insurance beyond malpractice, compared to 65% in the study group. Additionally, those with management training were more likely to visibly display patient rights, as required by law [68]. A significant difference was noted in assigning responsibility for supply management—61.7% in the study group vs. 27.6% in the control group (*p* < 0.01). Dentistry is known for its high stress levels, and workplace improvements could reduce burnout. In our study, 75% of respondents felt overwhelmed by non-clinical tasks. This aligns with findings by Jin et al. (2015), who emphasize the role of vocational motivation in managing burnout [69].

The data obtained from our study shows that all respondents faced difficulties when establishing their dental practices, and their knowledge of organizing and running the practice at the start of the activity was similar in both groups. Dentists who attended management courses showed a greater openness to using modern means of managing a dental practice: management software, collaboration with specialized companies for practice marketing, using the internet, and possessing tools to create a pleasant environment in the dental office compared to those in the control group. The market for effective management and marketing is based on the theory of influence and includes six key principles: reciprocity, commitment and consistency, social proof, authority, liking, and scarcity [70]. In relation to the economic, social, and dental market environment, modern companies’ marketing cannot be limited to the production and distribution processes but requires ongoing, complex communication with the external environment, which involves proper information sharing with leaders [71].

Many dentists may be unaware of their own leadership and management techniques and, as a result, the impact on their team members. Negative approaches can contribute to low morale and high staff turnover. In order to become more adept at applying certain approaches in given situations, dentists and their collaborators may need to undergo continuous professional development in leadership and management [72]. Effective and efficient management of the dental practice depends largely on all the factors described above, but we cannot exclude macroeconomic and political factors specific to our country [29].

## 5. Conclusions

This study highlights the significant impact of management education on the organizational and operational aspects of dental practices. The findings reveal that dentists who have participated in Health Management or Dental Practice Management courses are more likely to adopt modern organizational structures, such as limited liability companies (LLCs), compared to their peers who have not undergone such training, who predominantly operate individual medical practices (IMPs). This suggests that exposure to management training influences not only clinical practice but also strategic decisions regarding practice structure.

Furthermore, there is a clear trend indicating that with increased professional experience, dentists show a greater interest in acquiring management competencies. This reflects a growing recognition within the profession that clinical expertise alone is insufficient for the effective running of a dental clinic, and that managerial knowledge is essential for long-term sustainability and growth.

Importantly, the study shows that management courses shift the perception of leadership roles within dental practices. Dentists who attend these courses increasingly see the value of involving trained managers in overseeing practice operations. This insight opens the door to interdisciplinary collaboration and the professionalization of practice management.

Moreover, management education empowers dentists with practical skills in delegation and team coordination, contributing to a more efficient clinical workflow. By learning how to better manage administrative tasks and collaborate with other specialists, dentists can reduce workload stress and focus more on patient care.

Lastly, the results confirm that knowledge acquired in management training is not merely theoretical but is actively applied in daily practice. Dentists who have completed such courses report implementing specific management strategies to enhance the efficiency and quality of care within their clinics.

In summary, this study underscores the value of integrating management training into dental professional development. Not only does it support improved clinic performance and interdisciplinary collaboration, but it also promotes a forward-thinking approach to dental practice leadership, with potential implications for policy-making in dental education and continuing professional development programs.

## Figures and Tables

**Table 1:** Distribution of dentists according to demographic characteristics.

Variable	Study Group *n* (%)	Control Group *n* (%)	Total *n* (%)	*p*-Value
**Mean ± SD age**	39.25 ± 8.92	37.88 ± 10.38	38.48 ± 9.75	0.419
Group I	30 (50%)	44 (57.9%)	74 (54.4%)	-
Group II	28 (46.6%)	29 (38.2%)	57 (41.9%)	-
Group III	2 (3.4%)	3 (3.9%)	5 (3.7%)	-
**Gender**				
Male	22 (36.7%)	30 (39.5%)	52 (38.2%)	0.738
Female	38 (63.3%)	46 (60.5%)	84 (61.8%)	
**Dental office location**				
Urban	54 (90%)	56 (73.7%)	110 (80.9%)	0.160
Rural	6 (10%)	20 (26.3%)	26 (19.1%)	

SD = standard deviation.

**Table 2:** Distribution of doctors according to professional characteristics.

Variable	Study Group *n* (%)	Control Group *n* (%)	Total *n* (%)	*p*-Value
**Type of dental practice**				0.185
Private medical office (CMI)	22 (36.7)	33 (43.4)	55 (40.5)	
Limited liability company (SRL)	25 (41.7)	24 (31.6)	49 (36)	
Individual practice (PFI)	3 (5)	7 (9.2)	10 (7.4)	
Employed in private sector	9 (15)	6 (7.9)	15 (11)	
Employed in public sector	1 (1.6)	6 (7.9)	7 (5.1)	
**Years of professional experience**				0.069
<5 years	10 (16.7)	26 (34.2)	36 (26.5)	
5–10 years	17 (28.3)	16 (21)	33 (24.3)	
>10 years	33 (55)	34 (44.8)	67 (49.2)	
**Professional qualification**				0.411
General dentist	28 (46.7)	35 (46.1)	63 (46.3)	
Resident dentist	6 (10)	15 (19.7)	21 (15.4)	
Specialist dentist	16 (26.7)	17 (22.3)	33 (24.3)	
Senior dentist	10 (16.6%)	9 (11.9%)	19 (14%)	

**Table 3:** Respondents’ distribution based on their opinions regarding the best manager of a dental practice.

Variable	Study Group *n* (%)	Control Group *n* (%)	Total *n* (%)	*p*-Value
Dentist	29 (48.3)	50 (65.8)	79 (58.1)	0.041
Manager	31 (51.7)	26 (34.2)	57 (41.9)	

**Table 4:** The distribution of respondents based on the categories of collaborators of the dental office.

Variable	Study Group *n* (%)		Control Group *n* (%)		*p*-Value
	YES	NO	YES	NO	
Dentist	41 (68.3%)	19 (31.7%)	36 (47.4%)	40 (52.6%)	0.014
Orthodontist	46 (76.7%)	14 (23.3%)	48 (63.2%)	28 (36.8%)	0.090
Pediatric dentist	25 (41.7%)	35 (58.3%)	22 (28.9%)	54 (71.1%)	0.122
Prosthodontist	26 (4.3%)	34 (56.7%)	26 (34.2%)	50 (65.8%)	0.277
Endodontist	37 (61.7%)	23 (38.3%)	35 (46.1%)	41 (53.9%)	0.070
OMF surgeon	31 (51.7%)	29 (48.3%)	35 (46.1%)	41 (53.9%)	0.515
Dentoalveolar surgeon	32 (53.3%)	28 (46.7%)	35 (46.1%)	41 (53.9%)	0.399
Medical assistant	47 (78.3%)	13 (21.7%)	36 (47.4%)	40 (52.6%)	0.0002
Dental technician	48 (80%)	12 (20%)	63 (82.9%)	13 (17.1%)	0.665
Radiology technician	23 (38.3%)	37 (61.7%)	17 (22.4%)	59 (77.6%)	0.042
Front desk	26 (43.3%)	34 (56.7%)	14 (18.4%)	62 (81.6%)	0.002
Manager	20 (33.3%)	40 (66.7%)	14 (18.4%)	62 (81.6%)	0.046
Accountant	42 (70%)	18 (30%)	59 (77.6%)	17 (22.4%)	0.312
Cleaning staff	44 (73.3)	16 (26.7)	44 (57.9)	32 (42.1)	0.061

**Table 5:** The distribution of respondents based on their answers regarding the tasks of the medical assistant in the dental office.

Variable	Study Group *n* (%)		Control Group *n* (%)		*p*-Value
	YES	NO	YES	NO	
Schedules patients	42 (70%)	18 (30%)	46 (60.5%)	30 (39.5%)	0.251
Sterilizes the instruments	59 (98.3%)	1 (1.7%)	75 (98.7%)	1 (1.3%)	0.866
Prepares the medico-legal documents required for the therapeutic procedure	46 (76.7%)	14 (23.3%)	61 (80.3%)	15 (19.7%)	0.611
Prepares the patient’s treatment record	50 (83.3%)	10 (16.7%)	60 (78.9%)	16 (21.1%)	0.518
Prepares the dental office and necessary materials	58 (96.7%)	2 (3.3%)	73 (96.1%)	3 (3.9%)	0.850
Records treatments in the consultation register and in the patient file	46 (76.7%)	14 (23.3%)	59 (77.6%)	17 (22.4%)	0.894

**Table 6:** Distribution of respondents based on responses regarding the management of the dental practice.

Variable	Study Group *n* (%)		Control Group *n* (%)		*p*-Value
	YES	NO	YES	NO	
The existence of concerns for employee training	57 (95%)	3 (5%)	54 (71%)	22 (29%)	0.0003
Possession of a dental practice management program	35 (58.3%)	25 (41.7%)	13 (17.1%)	63 (82.9%)	*p* < 0.01
Difficulties encountered when establishing the dental practice	34 (56.7%)	26 (43.3%)	31 (40.8%)	45 (59.2%)	0.066
Knowledge regarding the organization and operation of the dental practice at the start of the activity	32 (53.3%)	28 (46.7%)	36 (47.4%)	40 (52.6%)	0.490
The existence of a strategic plan at the level of the dental practice	44 (73.3%)	16 (26.7%)	31 (40.8%)	45 (59.2%)	0.0001
The existence of a quality management plan for the dental office	46 (76.7%)	14 (23.3%)	30 (39.5%)	46 (60.5%)	*p* < 0.01
Periodic analysis of financial performance	38 (63.3%)	22 (26.7%)	37 (48.7%)	39 (51.3%)	0.088
The existence of a risk management plan for the dental practice	28 (46.7%)	32 (53.3%)	17 (22.4%)	59 (77.6%)	0.003
Possession of liability insurance for the dental practice	39 (65%)	21 (35%)	24 (31.6%)	52 (68.4%)	0.0001
Designation of a person responsible for supplying the dental practice with dental materials	37 (61.7%)	23 (38.3%)	21 (27.6%)	55 (72.4%)	*p* < 0.01
Possession of a contract with the National Health Insurance House (CNAS)	43 (71.7%)	17 (28.3%)	41 (53.9%)	35 (46.1%)	0.035
Display of patient rights in a visible location	49 (81.7%)	11 (18.3%)	57 (75%)	19 (25%)	0.352
Overloading due to secondary activities related to dental therapeutic procedures	48 (80%)	12 (20%)	54 (71%)	22 (29%)	0.232

**Table 7:** Distribution of respondents based on responses regarding modern means of managing the dental practice.

Variable	Study Group *n*(%)		Control Group *n*(%)		*p*-Value
	YES	NO	YES	NO	
Possession of dental practice management software	31 (51.7%)	29 (48%)	21 (27.6%)	55 (72.4%)	0.004
Collaboration with specialized companies for the marketing of the dental practice	23 (38.3%)	37 (61.7%)	13 (17.1%)	63 (82.9%)	0.005
Use of the internet in the dental practice	59 (98.3%)	1 (1.7%)	64 (84.2%)	12 (15.8%)	0.005
Possession of means to create a pleasant ambiance in the dental practice	56 (93.3%)	4 (6.7%)	60 (78.9%)	16 (21.1%)	0.019

## Data Availability

The original contributions presented in this study are included in the article. Further inquiries can be directed to the corresponding authors due to ethical reasons.

## Supplementary Materials

The following supporting information can be downloaded at: https://www.mdpi.com/article/10.3390/healthcare13131631/s1.
