# Supplementary material for: Integrated Dental Practice Management in Romania: A Cross-Sectional Case–Control Study on the Perceived Impact of Managerial Training on Efficiency, Collaboration, and Care Quality Dental
Source: Healthcare (Basel). 2025 Jul 7;13(13):1631. doi: 10.3390/healthcare13131631 (PMC12249282; doi:10.3390/healthcare13131631)
Supplement: Supplementary file 1 [file healthcare-13-01631-s001.zip › healthcare-3652106-supplementary.pdf]

QUESTIONNAIRE
Assessment of the Level of Implementation of Management Strategies in the Dental Office

This questionnaire is part of an academic research study regarding the management of dental offices. We kindly ask you to answer the questions below. Your responses are anonymous. Thank you!

1. **Age**

2. **Gender**
Mark only one oval
o Male
o Female

3. **Type of Dental Practice Organization**
Mark only one oval
o Individual Medical Office (CMI)
o Limited Liability Company (SRL)
o Independent Professional Practice (PFI)
o Employee in the private sector
o Employee in the public sector

4. **Location of the Dental Office**
Mark only one oval
o Urban
o Rural

5. **How long has it been since you graduated from university?**
Mark only one oval
o <5 years
o 5–10 years
o >10 years

6. **Which of the following categories do you belong to?**
Mark only one oval
o General Dentist
o Resident Doctor
o Specialist Doctor
o Senior Doctor

7. **Please select from the list below the categories of collaborators in your dental office:**
Mark only one oval per row

	YES	NO
General Dentist	o	o
Orthodontist	o	o
Pediatric Dentist	o	o
Prosthodontist	o	o
Endodontist	o	o
Maxillofacial Surgeon	o	o
Dentoalveolar Surgeon	o	o
Dental Assistant	o	o
Dental Technician	o	o
Radiology Technician	o	o
Receptionist - Front Office	o	o
Manager	o	o
Accountant	o	o
Cleaning Staff	o	o

8. **If you have a dental assistant, what are their responsibilities?**

Mark only one oval per row

Responsibility	YES	NO
Schedules patients	o	o
Sterilizes instruments	o	o
Prepares the legal-medical documents required for the therapeutic act	o	o
Prepares the patient's treatment chart	o	o
Prepares the office and necessary materials	o	o
Records treatments in the consultation register and in the patient's file	o	o

9. **Have you attended a Healthcare Management or Dental Office Management course so far?**
Mark only one oval
o Yes
o No

10. **Who do you think would best manage a dental office?**
o Dentist
o Specialized Manager

11. If you own a dental practice, did you encounter any difficulties when establishing it?
Mark only one oval
o Yes
o No

12. Do you sometimes feel overwhelmed due to activities secondary to the dental therapeutic act?
Mark only one oval
o Yes
o No

13. Do you use the Internet in your dental office?
Mark only one oval
o Yes
o No

14. Do you have a dental office management program?
Mark only one oval
o Yes
o No

15. Do you have practice management software for your dental office?
Mark only one oval
o Yes
o No

16. Have you designated someone responsible for supplying dental materials in the office?
Mark only one oval
o Yes
o No

17. Besides malpractice insurance, do you have civil liability insurance for your dental office?
Mark only one oval
o Yes
o No

18. Is there a strategic plan in place at your dental office?
Mark only one oval
o Yes
o No

19. Is there a risk management plan in your dental office?
Mark only one oval
o Yes
o No

20. Is there a quality management plan in your dental office?
Mark only one oval
o Yes
o No

21. Are the patients' rights displayed in a visible place?
Mark only one oval
o Yes
o No

22. Does the manager periodically conduct financial analyses in your dental office?
Mark only one oval
o Yes
o No

23. Is there an interest in staff training in your dental office?
Mark only one oval
o Yes
o No

24. When you began practicing as a dentist, did you know what was involved in organizing and running a dental office?
Mark only one oval
o Yes
o No

25. Do you have a contract with the Health Insurance House (Casa de Asigurări de Sănătate)?
Mark only one oval
o Yes
o No

26. Do you collaborate with specialized companies for your dental office's marketing?
Mark only one oval
o Yes
o No

27. Does your dental office include amenities that create a pleasant environment (TV, radio, music)?
Mark only one oval
o Yes
o No
